# How Safe Are Common Analgesics for the Treatment of Acute Pain for Children? A Systematic Review

**DOI:** 10.1155/2016/5346819

**Published:** 2016-12-18

**Authors:** Lisa Hartling, Samina Ali, Donna M. Dryden, Pritam Chordiya, David W. Johnson, Amy C. Plint, Antonia Stang, Patrick J. McGrath, Amy L. Drendel

**Affiliations:** ^1^Alberta Research Centre for Health Evidence, Department of Pediatrics, Faculty of Medicine and Dentistry, University of Alberta, Edmonton, AB, Canada; ^2^Department of Pediatrics, Faculty of Medicine and Dentistry, University of Alberta, Edmonton, AB, Canada; ^3^Women and Children's Health Research Institute, Edmonton, AB, Canada; ^4^Departments of Pediatrics and Physiology and Pharmacology, Faculty of Medicine, University of Calgary, Calgary, AB, Canada; ^5^Departments of Pediatrics and Emergency Medicine, University of Ottawa and Division of Emergency Medicine, Children's Hospital of Eastern Ontario, Ottawa, ON, Canada; ^6^Departments of Pediatrics and Community Health Sciences, University of Calgary and Alberta Children's Hospital Research Institute, Calgary, AB, Canada; ^7^IWK Health Centre and Science, Pediatrics, Psychiatry and Community Health and Epidemiology, Dalhousie University, Halifax, NS, Canada; ^8^Department of Pediatrics, Medical College of Wisconsin, Milwaukee, WI, USA

## Abstract

*Background*. Fear of adverse events and occurrence of side effects are commonly cited by families and physicians as obstructive to appropriate use of pain medication in children. We examined evidence comparing the safety profiles of three groups of oral medications, acetaminophen, nonsteroidal anti-inflammatory drugs, and opioids, to manage acute nonsurgical pain in children (<18 years) treated in ambulatory settings.* Methods*. A comprehensive search was performed to July 2015, including review of national data registries. Two reviewers screened articles for inclusion, assessed methodological quality, and extracted data. Risks (incidence rates) were pooled using a random effects model.* Results*. Forty-four studies were included; 23 reported on adverse events. Based on limited current evidence, acetaminophen, ibuprofen, and opioids have similar nausea and vomiting profiles. Opioids have the greatest risk of central nervous system adverse events. Dual therapy with a nonopioid/opioid combination resulted in a lower risk of adverse events than opioids alone.* Conclusions*. Ibuprofen and acetaminophen have similar reported adverse effects and notably less adverse events than opioids. Dual therapy with a nonopioid/opioid combination confers a protective effect for adverse events over opioids alone. This research highlights challenges in assessing medication safety, including lack of more detailed information in registry data, and inconsistent reporting in trials.

## 1. Introduction

Pain is the most common reason for seeking healthcare in the Western world [1–3]. Key organizations have voiced concern with our medical services' inability to provide appropriate analgesia for children's pain [4–8]. Inadequate pain treatment can have significant detrimental effects [9–12]. Still, fear of adverse events (AEs) is commonly cited by both families and physicians as an obstruction to appropriate use of analgesic medication in childhood [13, 14].

AEs are a major health problem for affected children, their family, and society [15–17]. Surprisingly, comprehensive drug safety in children remains understudied, despite emerging evidence that AEs are frequent and are commonly cited as a reason for terminating prescribed therapy [15, 16]. A systematic review of AEs in hospitalized pediatric patients reported an overall incidence rate of 9% [18]. Furthermore, up to 30–50% of pediatric analgesic users will experience* at least* one AE [13]. Milder AEs (i.e., vomiting, sleepiness, and constipation) contribute to limitations in activity and function for children and negatively impact their caregivers' productivity and time off work [14]. Importantly, AEs may discourage future use of analgesics, thereby exposing children to unnecessary pain and its resultant negative consequences.

Children's pain management varies greatly across North America [14, 19]. Recently, clinicians have been compelled to rapidly shift their prescribing practices due, in part, to concerns regarding the safety profile of oral opioids [14, 20–24]. With the FDA boxed warning regarding codeine use in children post-ENT surgery [23] as well as the European Medicines Agency and Health Canada's advisory to avoid all codeine use in children less than 12 years of age [24, 25], clinicians have reduced their use of codeine and now search for a suitable alternative oral opioid. A recent survey of North American physicians showed considerable variability in practice management by center, country, and specialty [26]. The American Academy of Pediatrics' consensus statement on the assessment and management of pain in children recommends acetaminophen, ibuprofen, and opioids as the top three medication choices for the treatment of acute pain in children [4]. These are also the top three most commonly used treatments in the emergency department for children with fracture pain [14, 22, 26–28]. It stands to reason that clinicians (and certainly patients and their families) would prefer the drug that has the best safety profile. However, as noted in the 2014 report by the Council of Canadian Academies, available published literature is not properly synthesized to provide the data needed to make such treatment decisions [29].

This systematic review compares the safety profiles of three groups of oral medications, acetaminophen, nonsteroidal anti-inflammatory drugs (NSAIDS), and opioids, to manage acute, nonsurgical pain in children treated in ambulatory settings. While much has been written about the efficacy of these medications, to date, there has been no comprehensive synthesis of safety and in particular their comparative safety. Moreover, since emerging studies are suggesting equivalence (or close to equivalence) and clinical equipoise for many of the current pain management options [13, 19, 30, 31], clinicians may make practical decisions driven by the perceived safety profile. In an effort to address known challenges in safety literature nomenclature [32], we attempted to capture a range of undesirable effects (e.g., side effects, adverse effects, adverse events, and adverse reactions). For the purposes of this manuscript, we use the term “adverse events” to represent all safety concerns captured by this variable nomenclature.

## 2. Methods

We followed a protocol established a priori (available from authors) based on standards for conducting and reporting systematic reviews [33–35].

### 2.1. Search

A research librarian searched (from inception to July 2015) the Cochrane Central Register of Controlled Trials (CENTRAL), MEDLINE (Appendix  A), EMBASE, International Pharmaceutical Abstracts, TOXNET, BIOSIS Previews, PubMed, and Web of Science. We searched relevant conference proceedings and abstracts from the American Pain Society (2011–2015), Canadian Pain Society (2011–2015), International Symposium of Pediatric Pain (2015), North American Congress of Clinical Toxicology (2011–2015), and the European Association of Poison Centers and Clinical Toxicologists (2011–2015). We also searched clinicaltrials.gov and the Australian New Zealand Clinical Trials Registry. We checked reference lists of relevant studies and searched websites of regulatory agencies. We contacted the U.S. Food and Drug Administration (FDA) and Health Canada for safety data related to the medications of interest. For all included studies we searched to see if they had been cited by new, relevant studies using Web of Knowledge and Google Scholar. Searches were not restricted by language or publication status.

### 2.2. Study Selection

Search results were screened independently by two reviewers. Two reviewers then assessed the full text of all potentially relevant citations using a standard form with predefined eligibility criteria. Disagreements were resolved by consensus.

### 2.3. Inclusion Criteria

We included primary studies of any design involving children (<18 years) with acute pain (pain related to injury or illness less than 3 months in duration) who were treated in an ambulatory setting (e.g., outpatient clinics, emergency). The ambulatory setting was chosen as it represents the most common location for presentation of children with acute injuries and illnesses requiring short-term analgesic use. Pain medications included acetaminophen, NSAIDS, and opioids. We included any study design and publication type. We excluded studies of surgically induced pain, as we felt these patients represented medically induced pain and included the possible influence of general or regional anesthesia.

### 2.4. Data Extraction

One reviewer extracted data using a structured form; a second reviewer verified data for accuracy and completeness. We extracted study and patient characteristics; interventions (type, dose, route of administration, timing, and duration); care setting (e.g., emergency department, outpatient clinic, primary care, and others); AEs; and funding source.

We extracted AEs as reported in the studies and grouped them into nine categories: nausea, vomiting, other gastrointestinal symptoms, headache, drowsiness (includes sleepiness and tiredness), dizziness, other CNS symptoms, dermatological symptoms, and pulmonary symptoms. For each AE, we counted each event as if it corresponded to a unique individual.

### 2.5. Assessment of Methodological Quality

We used the McMaster Quality Assessment Scale of Harms, a validated tool covering issues of how data on harms were defined, collected, and reported. Two reviewers independently assessed quality and resolved discrepancies through discussion.

### 2.6. Data Synthesis

A description of the studies is provided in tables. We present a summary of AEs by treatment arm (i.e., intervention) for an overall picture of which interventions had a high risk of specific AEs. This enabled us to include data from both comparative and noncomparative studies. For each AE, risks (incidence rates) were pooled using a random effects model to obtain a summary estimate and 95% confidence interval (CI). Analyses were conducted using Review Manager 5.2 (Cochrane Collaboration, 2012).

## 3. Results

**Figure 1:**
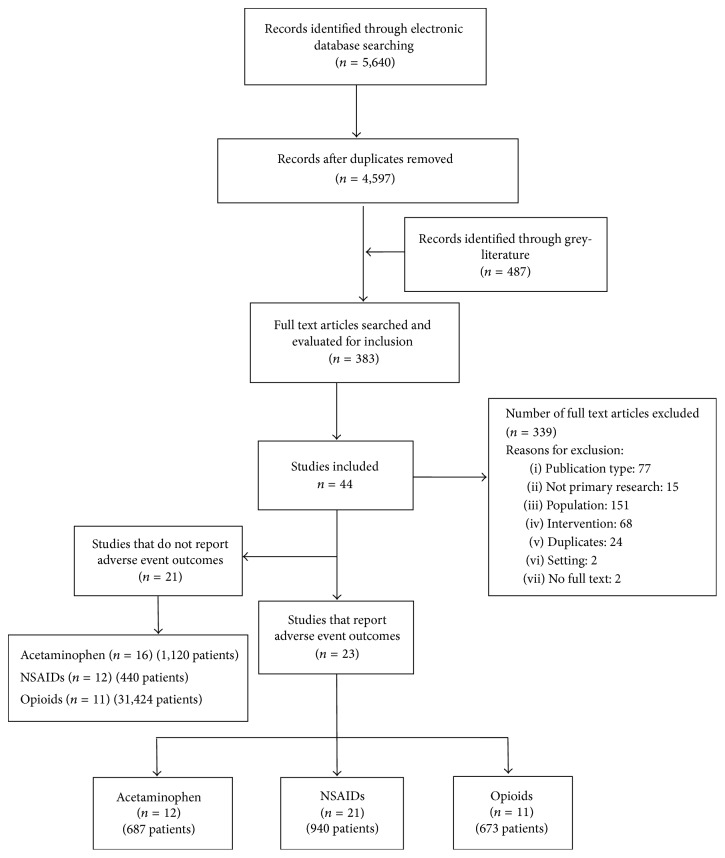
PRISMA flow diagram of study retrieval and selection.

Forty-four studies met our inclusion criteria; however, 21 did not report AEs (Figure 1) [36–56]. Therefore, we included 23 studies involving 2,300 patients: 17 randomized controlled trials, 2 nonrandomized controlled trials, 1 case report, 1 cross-sectional survey, 1 chart review, and 1 prospective cohort (Table 1) [13, 19, 27, 57–76]. Studies were published between 1991 and 2014 (median year 2007) and were conducted in the United States (*n* = 7), Canada (5), France (3), Italy (3), and Germany (2) and one each in Finland, New Zealand, and the United Kingdom. Most studies did not report on sources of funding (*n* = 13), one specifically reported no funding [68], seven reported grant funding [13, 19, 62, 64, 66, 69, 75, 76], and two reported funding from industry [70, 75].

The median quality score was 8/14 (interquartile range 6 to 8) (Appendix  B). Only 41% predefined harms while none defined serious or severe AEs. Sixty-nine percent included active data collection for AEs; 48% involved passive (six studies used both). Seventy-eight percent specified who collected AE data, and 57% provided information on their training and/or background. Forty-three percent specified the timing and frequency of AE collection; only 9% used a standard scale or checklist for harms collection. The majority (83%) specified that the reported harms encompassed all events (not a select sample). The majority specified the number of AEs in each study arm (90%), the number that withdrew or were lost to follow-up for each group (87%), and the number for each type of AE (78%). Few specified the type of analyses undertaken for harms data (35%).

**Figure 2:**
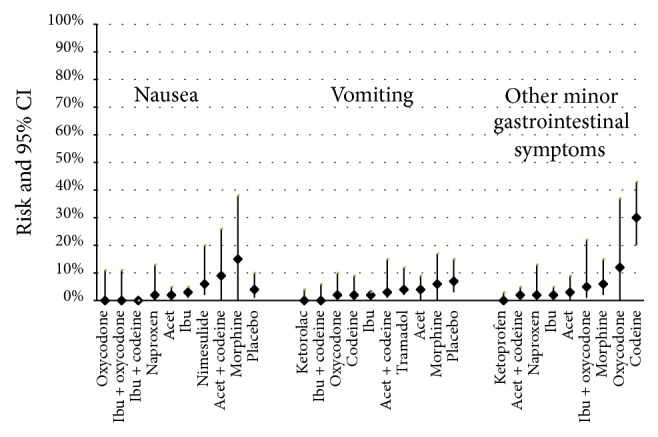
Risk (incidence rate) of nausea, vomiting, and other minor gastrointestinal symptoms by medication. ^*∗*^Other minor gastrointestinal (GI) symptoms included diarrhea, gastric pain, pills being hard to swallow, feeling sick, being unwell with vomiting and reduced urine output, emesis, constipation, sore stomach and abdominal pain, dry mouth, and unspecified GI symptoms. Acet = acetaminophen; ibu = ibuprofen. Results are presented in lowest to highest risk order with placebo at the end (where data were available).

**Figure 3 fig3:**
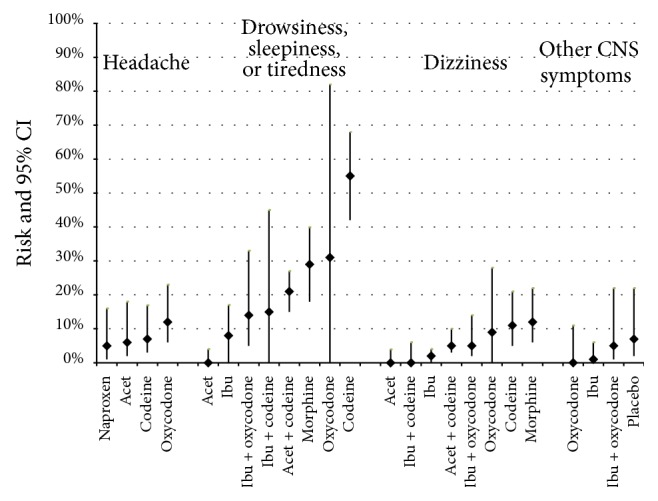
Risk (incidence rate) of headache, drowsiness, dizziness, and other minor central nervous system symptoms by medication. ^*∗*^Other minor central nervous system (CNS) symptoms included being lightheaded, agitation, twitchiness, and unspecific CNS symptoms. Results are presented in lowest to highest risk order with placebo at the end (where data were available).

**Figure 4:**
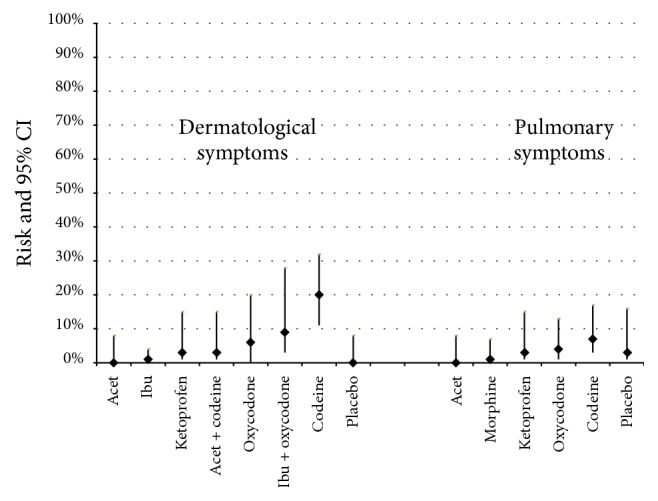
Risk (incidence rate) of dermatological and pulmonary symptoms by medication. ^*∗*^Dermatological symptoms included itchiness, rash, and pruritus. Results are presented in lowest to highest risk order with placebo at the end (where data were available).

Figures 2 [Fig fig3]–4 present the risks of AEs for all pain medications and placebo (Appendix  C provides detailed risk data). The following summarizes the data by type of AE.

*Gastrointestinal (GI) (Figure 2).* Acetaminophen had a similar GI AE profile as NSAIDS. Opioids trended towards greater “other GI AEs,” including constipation. Codeine monotherapy showed cumulatively more GI AEs than all other analgesics. NSAIDS and acetaminophen reported less than 10% rate of GI AEs. Opioid/nonopioid combinations had varying degrees of GI AEs associated with them; of note, oral morphine demonstrated the highest reported risk of nausea, followed by acetaminophen with codeine combination medication. Placebo-related AEs of nausea and vomiting were equal to or greater than that of some pain medications.

*Central Nervous System (CNS) (Figure 3).* Opioid monotherapy showed the highest risk of CNS AEs, with drowsiness/tiredness being noted in close to one-third of children receiving oxycodone or oral morphine and half of children receiving codeine. CNS symptoms of drowsiness and dizziness were notably higher for all opioid medications, when compared to nonopioid choices. Oxycodone and oral morphine have comparable risks for both drowsiness and dizziness. Opioid/nonopioid combination medications had a lower risk of CNS AEs.

*Dermatological and Pulmonary System (Figure 4).* Opioid medications demonstrated a greater risk of dermatologic symptoms. Children receiving only codeine had almost double the risk of experiencing dermatologic manifestations compared to all other medications. Pulmonary AEs were rare. No children receiving a NSAID experienced bronchospasm.

## 4. Registry Data 

We requested acetaminophen data from the U.S. Food and Drug Administration (FDA) through the U.S. FDA Adverse Event Reporting System. The report provided was a nonsearchable PDF comprising 7,523 pages with 17,806 reports of AEs for acetaminophen for all ages (i.e., adults and children). The team confirmed with the FDA that it was not possible to restrict the search results by age group or intention (i.e., nonintentional causes). Consequently, it was not feasible to include FDA data in this manuscript.

Through Health Canada, we received reports of AEs among children and youth from the Canada Vigilance Adverse Reaction Online Database. We received a total of 625 reports for all the three classes of drugs. For acetaminophen, we identified 232 reports; 39 were relevant based on our inclusion criteria. The median age was 5 years (range 7 weeks to 18 years). The most common AEs were vomiting (*n* = 8), dermatological symptoms (*n* = 7), other GI symptoms (*n* = 5), and psychiatric effects (*n* = 4). The remaining AEs were reported for either one or two patients. For NSAIDS, we identified 209 reports; 69 were relevant. The median age was 7 years (range 3 months to 18 years). The most common AEs were allergic-type reactions (*n* = 14), headache (*n* = 4), vomiting (*n* = 4), nausea (*n* = 2), other GI symptoms (*n* = 4), dermatological (*n* = 3), and psychiatric effects (*n* = 3). For opioids, we identified 184 reports; 21 were relevant. The median age was 13 years (range 18 months to 18 years). The most common AEs were other GI symptoms (*n* = 4), vomiting (*n* = 3), nausea (*n* = 3), and dermatological (*n* = 3).

Four deaths were reported in the Health Canada data (Table 2). Case  1 was a 2-year-old who took morphine (route of administration unclear) and suffered respiratory failure. Case  2 was an 18- year-old who took acetaminophen, alone, as well as an acetaminophen/codeine combination. The route of administration for both was oral; no dosing information was provided. Case  3 was a 4-year-old who took an acetaminophen/codeine combination (oral; no dosing information). The patient was described as having a “medically important condition” but no further description was provided. Respiratory distress was plausible with codeine use. Case  4 was a 3-month-old, 4 kg child who was given acetaminophen. Reaction information included apnea, cyanosis, and respiratory depression, which are not typical AEs of acetaminophen. Drug levels were noted to be supratherapeutic but no further details were provided; a 4 kg weight likely suggests prematurity or chronic illness.

## 5. Discussion

Each year, more than 50% of children use at least one medication [77], with acetaminophen and ibuprofen being the most common. There is an alarming paucity of research regarding the drug safety of common analgesics in children. Children's response to medications is often different from that of adults due to metabolism, ontogeny, and other age-related differences; as such, examination of safety data must be specific to children. Our study is one of the first to synthesize the currently available evidence and provides urgently needed safety information for clinicians treating children with acute pain in ambulatory settings.

Our study of the safety profiles of commonly used pain medications for children in the outpatient setting has demonstrated that (1) acetaminophen and ibuprofen have similar risk of nausea and vomiting, (2) opioids have the greatest risk of CNS AEs, and (3) dual therapy with a nonopioid/opioid medications results in a lower risk of AEs than opioids alone.

Our findings, specific to its use for acute, painful conditions, are consistent with previous work that has examined acetaminophen and ibuprofen in the context of its antipyretic use. A large randomized controlled trial (RCT) of ibuprofen and acetaminophen in outpatient practice showed the risk of hospitalization for gastrointestinal bleeding, renal failure, or anaphylaxis was not increased following short-term use of ibuprofen [78]. A notable limitation of their study was the lack of information on less severe outcomes. A second large RCT of febrile infants (<2 years of age) receiving acetaminophen or ibuprofen demonstrated a 1.4% risk of serious AEs (defined as rate of admission for acute gastrointestinal bleeding, acute renal failure, anaphylaxis, Reye's syndrome, asthma, bronchiolitis, and vomiting/gastritis) [79]. A 2004 systematic review of acetaminophen and ibuprofen, consisting of 3 pain studies and 14 fever studies in children, concluded that single doses of ibuprofen and acetaminophen had similar safety profiles, when assessing “major” (e.g., abdominal pain, vomiting, and hypothermia) and “minor” (e.g., nausea, sweating, and cutaneous rash) AEs [80]. Our study adds important and* comprehensive* information regarding AEs for healthy children with injury or illness-related pain. This group is medically different from febrile and infected children and, as such, merits separate consideration.

Determining the appropriate analgesic agent for a child with acute pain can be a complex decision, influenced by multiple factors including patient age and genomics, anticipated degree and duration of pain, ability to swallow pills, and preexisting medical problems. An understanding of the AE profile can aid in making an evidence-based, personalized choice that takes into account a child's individual circumstances.

We have determined that acetaminophen, traditionally considered one of the most widely studied, gastrointestinally “benign” pain medications (as compared to NSAIDs or opioids), has a similar GI side effect profile as ibuprofen, for children. As such, it would make clinical sense to use the pain medication that conferred the most clinical analgesic effect, even when GI side effects were of concern for a specific child. Limiting short-term ibuprofen use due to concern for GI AEs is not supported by current evidence. It is noteworthy that the placebo-related side effects of nausea and vomiting were equal to or greater than that of the various pain medications. This phenomenon has been previously well-described [81] and underscores the importance of measuring and comparing relative differences in side effect profiles, rather than only absolute values. Further, in the context of treating pain, one must be cautious to attribute all AEs to the pain medications used, as pain, itself, may plausibly have a causative role in some of the symptoms (e.g., nausea, agitation).

Oxycodone, oral morphine, and codeine monotherapy had the greatest risk of CNS side effects, with drowsiness/tiredness being noted in close to one-third of children receiving oxycodone or oral morphine and half of children receiving codeine. CNS symptoms of drowsiness and dizziness were notably higher for all opioid medications, when compared to nonopioid choices. This makes physiologic sense, as opioids have CNS depressant effects which NSAIDS and acetaminophen lack. A child who needs to avoid CNS symptoms (e.g., a school-aged child, during daytime hours) should likely be treated with ibuprofen or acetaminophen for mild-moderate pain, as they have a lower AE profile, as compared to low-mid dose opioids, a comparably potent analgesic agent [19, 27, 66]. Current evidence suggests that ibuprofen likely has superior analgesic potency for many clinical conditions including fractures, sprains, and postoperative pain [13, 19, 76, 81–84]. Further, knowing that its GI AE profile is comparable to acetaminophen, one would likely choose ibuprofen as first line therapy for most such pediatric acute pain conditions with mild to moderate pain. When escalating pain necessitates the use of oral opioids, dual therapy (with a nonopioid medication, such as ibuprofen or acetaminophen) appears to “protect” the child against many of the negative AEs associated with opioids, particularly CNS effects. This is likely due to decreased opioid dosage required to achieve analgesia. Opioid medications should be added to, rather than replace, these nonopioids when needed for moderate-severe pain, a strategy endorsed by the World Health Organization [85].

Opioid medications were associated with a greater risk of dermatologic symptoms, which is plausible given the histamine release phenomenon that can occur with their use [86]. Children receiving only codeine had almost double the risk of experiencing dermatologic manifestations compared to all other medications. Of note, however, the confidence intervals for all opioid mono- and combination therapies overlapped significantly, with the notable exception of codeine monotherapy; children receiving only codeine had almost double the risk of experiencing dermatologic manifestations. Pulmonary side effects were rare. No children in the included studies receiving an NSAID experienced bronchospasm, a suggested risk for ibuprofen in some medical literature pertaining to fever management [87, 88].

Codeine monotherapy has cumulatively more gastrointestinal side effects than all other analgesics that were a part of this review, including other opioids. GI AEs associated with codeine monotherapy are poorly tolerated by children and may result in premature termination of therapy [13, 89] and significant short- and long-term effects [90–92] due to untreated pain. This information, coupled with the recent FDA warning [93] and other recommendations [24, 25, 94] to avoid codeine use in children under 12 years of age, supports the general avoidance of codeine use in opioid-naïve children, except in the exceptional circumstance of an older child who has previously received codeine and tolerated it well.

This study has also led to some noteworthy observations regarding the use of national databases for medication-related research. We contacted the FDA through the Adverse Event Reporting System and received a report that was not usable for study purposes. We contacted Health Canada for data collected through the Canada Vigilance Adverse Reaction Online Database, and reports were readily available to us. While we were able to review them for relevance, it was impossible to draw conclusions regarding causality. As such, the results should be interpreted cautiously as there are several limitations: there is no denominator information to calculate incidence rates; reports are voluntarily submitted by consumers and nonphysician health professionals and lack details. We recommend that federal agencies consider modifying the manner in which medication-related AEs are reported and recorded. This would allow researchers and policy-makers to understand and use this information in a more meaningful way. Specifically, we suggest recording the treating clinicians' postulated interpretation of the cause of the AEs, creation of a searchable database, and clearer recording of the dose and frequency of the drugs used.

Given the inconsistent and often passive information collection of AEs in past studies, it is imperative that future trials of pain medications include a standardized and universal approach to the collection of AEs, in order to create a growing repository of such information, which can be resynthesized at a future date, and lead to even more robust conclusions regarding safety.

## 6. Strengths and Limitations 

To our knowledge, this is the first clinician-friendly, comprehensive synthesis of AEs for the most commonly used pain medications for children. This is an important first step in addressing the lack of postmarket safety data for medications for children. Our unique style of presenting the data, based on AE rather than medication type, provides a more clinically meaningful way to interpret the results, as clinicians generally approach decision-making for patients from the perspective of “Which medication will provide the most pain relief with the least symptoms of concern for my patient?”

This study has some limitations. The synthesis of information was dependent on accurate and explicit recording of AEs within the original studies. Many of the included studies were not designed to systematically collect data on adverse events. As such, our results are a reflection of what is available but may not exactly reflect the true postmarket safety of the included medications. Further, the number of studies and patients is small given the prevalent use of these agents in practice. In some cases, the rates were variable across studies with wide confidence intervals. This could be due to a number of factors (e.g., dosing); however, few studies and sparse data did not allow for subgroup analyses to delineate risks according to other factors. Data was particularly sparse for NSAIDs other than ibuprofen. Results and conclusions regarding ibuprofen should not be extrapolated to other NSAIDs that may have more potent adverse event profiles. Finally, we were not able to interpret the FDA and Health Canada data in a meaningful fashion.

## 7. Conclusion

Based on the available evidence, ibuprofen and acetaminophen have similar reported adverse effects and notably less adverse events than opioids, for the initial treatment of acute pain in children in ambulatory settings. Dual therapy with a nonopioid/opioid combination confers a protective effect for AEs over opioids alone, suggesting that opioid medications should be added to, rather than replace, nonopioids when needed for moderate-severe pain. In order to allow for meaningful synthesis and evidence-informed care, it is imperative that future trials of pain medications include a standardized and universal approach to the collection of AEs and that ongoing national data registry monitoring be more detailed surrounding AEs (particularly causation).

## Supplementary Material

Appendix A: Search strategy for Medline. Appendix B: Detailed risk data by study and adverse event. Appendix C: This file contains study level details for each of the AEs described, including the dosage, the number of events, the sample size, and the risk.

## Figures and Tables

**Table 1:** Characteristics of included studies (in chronological order by date of publication).

Author, year country, funding source	Study design	Setting age, range (years)	Painful condition studied	Comparisons (*n* = number of subjects/patients)	AE defined a priori by authors^*∗*^	Author conclusions
Bertin et al., 1991, France, NR	RCT	ED mean 7.95 (SD 1.85)	Acute tonsillitis and pharyngitis (duration of sore throat ≤ 48 h)	(i) Ibuprofen (10 mg/kg), *n* = 77 (ii) Acetaminophen (10 mg/kg), *n* = 78 (iii) Placebo (10 mg/kg), *n* = 76	Nausea, abdominal pain, cutaneous rash	Ibuprofen, combined with antibiotic therapy, is effective and well-tolerated short-term treatment for pain

Bertin et al., 1996, France, NR	RCT	Outpatient clinic 1–6.75 (mean 2.98 ± 1.33)	Otoscopically proven acute otitis media, either unilateral or bilateral	(i) Ibuprofen (10 mg/kg), *n* = 71 (ii) Acetaminophen (10 mg/kg), *n* = 73 (iii) Placebo, *n* = 75	Nausea, vomiting, abdominal pain, cutaneous rash	11 children experienced mild unexpected events; treatment was never interrupted because of unexpected events

Hämäläinen et al., 1997, Finland, NR	RCT	ED NR	Migraine (≥2 migraine attacks/month lasting ≥2 h)	(i) Acetaminophen (15 mg/kg), *n* = 88 (ii) Ibuprofen (10 mg/kg), *n* = 88 (iii) Placebo: cellulose (NR), *n* = 88	Nausea, vomiting, gastric pain	No significant difference in number of AEs; acetaminophen and ibuprofen are effective and well-tolerated for acute migraine, with acetaminophen having faster onset but slightly less effect and ibuprofen giving best relief

Pothmann and Lobisch, 2000, Germany, NR	RCT, cross-over	Ambulatory clinic + home 6–12	Tension-type headache (IHS criteria)	(i) Flupirtine (6–8 years = 50 mg, 9–12 y = 100 mg) (ii) Acetaminophen (6–8 y = 250 mg, 9–12 y = 500 mg), *n* = 19	NR	Relevant side effects could not be observed

Soriani et al., 2001, Italy, NR	NRCT	ED mean 12.8 (SD 3.1)	Migraine (≥6-month duration and ≥1 attacks/month)	(i) Acetaminophen (15 mg/kg), *n* = 33 (ii) Nimesulide (2.5 mg/kg), *n* = 33	Mild abdominal discomfort, nausea	No significant difference in side effects; nimesulide associated with low occurrence of ADRs especially in GI tract; effects in liver are within or below the general incidence with other NSAIDs

Lewis et al., 2002, USA, NR	RCT	Home 6–12	Migraine (IHS-R criteria)	(i) Ibuprofen (7.5 mg/kg), *n* = 69 (ii) Placebo, *n* = 69	Nausea, vomiting, photophobia, phonophobia	Children's ibuprofen suspension (7.5 mg/kg) is effective and well-tolerated for pain relief in acute treatment of migraine, particularly in boys.

Tanabe et al., 2002, USA, grants from multiple nonindustry sources	NRCT	ED 5–17	Minor extremity trauma including and distal to elbow and knee	(i) Standard care (ice, elevation, and immobilization), *n* = 26 (ii) Standard care + distraction (e.g., guided imagery, music therapy, reading, or being read to), *n* = 24 (iii) Standard care + ibuprofen (10 mg/kg), *n* = 26	NR	One child experienced nausea after taking ibuprofen

Wille et al., 2005, France, NR	Prospective cohort (single arm)	ED 0.5–6	Injuries to extremities with deformation; burns requiring hospitalization	Morphine (0.5 mg/kg), *n* = 91	Nausea, vomiting, decrease in oxygen saturation, drowsiness	No serious AEs and few minor side effects; oral morphine monotherapy can be given within an analgesic protocol with few minor side effects

Drendel et al., 2006, USA, NR	Cross-sectional survey	Hospital-based pediatric orthopedic clinic 1–18	Isolated extremity fracture receiving follow-up care at orthopedic clinic within 10 days of injury	Patients (*n* = 98) received ibuprofen, acetaminophen + codeine, acetaminophen, other narcotics (hydrocodone, oxycodone, and rofecoxib), or a combination of ibuprofen, acetaminophen, and acetaminophen + codeine; 6 patients received no pain medication; 3 were unsure	“Other” effects from the medication	AEs not reported by drug; those receiving acetaminophen + codeine appeared to experience more other effects. Physicians and caregivers may have concerns about these side effects when dosing the medication

Evers et al., 2006, Germany, NR	RCT	Outpatient clinic 6–18	Migraine with and without aura	(i) Zolmitriptan (2.5 mg), *n* = 29 (ii) Ibuprofen (200 mg for children <12 or 400 mg for adolescents), *n* = 29 (iii) Placebo, *n* = 29	Dizziness, somnolence, gastrointestinal	No serious AEs occurred; all AEs were mild and resolved completely; zolmitriptan but not ibuprofen produced significantly more AEs than placebo

Clark et al., 2007, Canada, Hospital Research Institute	RCT	ED 6–17	Musculoskeletal injury (to extremities, neck, or back) occurring within 48 h	(i) Codeine (1 mg/kg, max 60 mg), *n* = 112 (ii) Acetaminophen (15 mg/kg, max 650 mg), *n* = 112 (iii) Ibuprofen (10 mg/kg, max 600 mg), *n* = 112	Minor AEs such as nausea, sleepiness, constipation	AEs were minimal; no significant difference between groups for minor AEs (nausea, sleepiness, constipation)

Cukiernik et al., 2007, Canada, Health Research Institutes	RCT	ED 8–14	Isolated soft tissue injury of ankle (no history of gastric ulcers or allergy to acetaminophen or naproxen)	(i) Naproxen (20 mg/kg), *n* = 41 (ii) Acetaminophen (15 mg/kg), *n* = 36	NR	No significant difference in efficacy or AEs. Given acetaminophen's wider safety margin and a large experience in children, acetaminophen may be the preferred agent for therapy of soft tissue injury in children and adolescents

Ismail et al., 2007, UK, NR	Case report	GP clinic 8	2-day history of neck and back pain	(i) Acetaminophen (500 mg) and ibuprofen (200 mg), *n* = 1	NA	Ibuprofen is generally safe and effective as antipyretic and analgesic; risk of acute papillary necrosis, particularly in dehydrated children

Koller et al., 2007, USA, Academic	RCT	ED	Suspected orthopedic injury; patients with baseline score of ≥4 on faces pain scale	(i) Oxycodone (0.1 mg/kg), *n* = 22 (ii) Ibuprofen (10 mg/kg), *n* = 22 (iii) Oxycodone + ibuprofen (0.1 mg/kg, 10 mg/kg), *n* = 22	NR	Oxycodone or ibuprofen monotherapy avoids the increase in AEs more than when given together

Charney et al., 2008, USA, NR	RCT	ED	Suspected isolated forearm fractures	(i) Oxycodone (0.2 mg/kg), *n* = 51 (ii) Codeine (2 mg/kg), *n* = 56	Vomiting, headache, dizziness, difficulty walking, difficulty breathing, tiredness, abdominal pain, itching, sweating, dry mouth	Minor AEs in both groups; itching less in oxycodone group

Drendel et al., 2009, USA, hospital foundation	RCT	ED 4–18	Fracture of radius, ulna, or humerus (no open fracture)	(i) Acetaminophen + codeine (120 mg/5 mg per 5 mL), *n* = 167 (ii) Ibuprofen (100 mg/5 mL), *n* = 169	Nausea, vomiting, drowsy, dizzy, constipation, other	Children receiving ibuprofen had significantly fewer AEs; both children and parents were more satisfied with ibuprofen; significantly higher rates of nausea and vomiting for children receiving acetaminophen + codeine

Friday et al., 2009, USA, NR	RCT	ED	Isolated extremity injury with tenderness to palpation from clavicle or femoral neck to distal phalanges; pain score ≥ 5 (of 10) at triage	(i) Acetaminophen + codeine (1 mg/kg, max 60 mg), *n* = 34 (ii) Ibuprofen (10 mg/kg, max 400 mg), *n* = 34	Vomiting, pruritus, nausea	AEs were minimal

Shepherd and Aickin, 2009, New Zealand, no funding	RCT	ED 5–14	Fracture management within 24 h of injury; acute, nonpathological fracture of distal humerus, any part of radius, ulna, tibia, or fibula; patient able to be managed as outpatient	(i) Acetaminophen (15 mg/kg), *n* = 54 (ii) Ibuprofen (10 mg/kg), *n* = 40	Vomiting, tiredness, dizziness	Parent-reported sleep quality did not differ between groups; no significant differences in side effects between groups

Richer et al., 2010, Canada, hospital foundation, national health funding agency	Chart review	ED 5–17	Migraine (headache at time of physician assessment and diagnosis of migraine by emergency physician)	(i) Ibuprofen (various doses) (ii) Acetaminophen (various doses) 1694 patients in study	NA	No serious AEs; dystonia, agitation, hypotension, and paresthesias were observed in association with a dopamine receptor antagonist

Ruperto et al., 2011, Italy, industry, research agency	RCT	ED 6–12	Pharyngotonsillitis	(i) Acetaminophen (12 mg/kg corresponding to 1 mL/2 kg), *n* = 34 (ii) Ketoprofen lysine salt (40 mg), *n* = 33 (iii) Placebo, *n* = 32	Bronchitis, rash, diarrhoea, cough	No serious AE; no events were related to the drugs or placebo

Le May et al., 2013, Canada, hospital research centre, national agency, industry	RCT	ED 7–18	Musculoskeletal injury to limb within 72 h; pain score > 3 on 0–10 VAS; bony tenderness, swelling, limited ROM, or angulation < 30°	(i) Codeine (1 mg/kg, max 60 mg) + Ibuprofen (10 mg/kg, max 600 mg), *n* = 42 (ii) Ibuprofen + placebo (10 mg/kg, max 600 mg), *n* = 41	Drowsiness, nausea, vomiting, dizziness	1 patient in experimental group with nausea; no other side effects or AEs reported in either group; side effects were minimal

Neri et al., 2013, Italy, NR	RCT	ED 4–17	Suspected fracture or dislocation	(i) Ketorolac (0.5 mg/kg, to a max of 20 mg (= 0.025 mL/kg of the solution, maximum 1 mL), *n* = 64 (ii) Tramadol (2 mg/kg, to a max of 100 mg, 0.020 mL/kg mL of the solution, and maximum of 1 mL), *n* = 67 (iii) Placebo, *n* = unclear	Vomiting, vomiting, dry mouth	No significant differences in AEs; fewer side effects reported in ketorolac group

Poonai et al., 2014, Canada, university research grant	RCT	ED 5–17	Nonoperative, radiographically evident extremity fracture	(i) Morphine (0.5 mg/kg, to a max of 10 mg), *n* = 66 (ii) Ibuprofen (10 mg/kg, to a max of 600 mg), *n* = 68	NR	No severe adverse drug reactions (e.g., immune-mediated hypersensitivity) were reported by any of the participants, and there were no deaths. Significantly more participants in the morphine group had adverse effects, the most common of which was drowsiness

^*∗*^Only five studies described how AEs were measured: Bertin et al. 1991 (on basis of clinical criteria); Evers et al. 2006 (patients or parents recorded adverse events at baseline and at 0.5, 1, 2, 4, and 24 hours after drug intake); Wille et al. 2005 (evaluated by nurse); Drendel et al. 2006 (26-question survey from patient's caregiver); Drendel et al. 2009 (caregivers and their children used diary to record tolerability of assigned medication daily).

**Table 2:** Description of deaths reported in Health Canada's Vigilance Adverse Reaction Online Database.

Age	Source of report	Medication	Other relevant information
2 years	Nonhealth professional	Morphine (no route or dosing information)	Respiratory failure was reported
18 years	Physician	Tylenol and acetaminophen plus codeine (both oral; no dosing information)	None reported
4 years	Nonphysician health professional	Acetaminophen plus codeine (oral; no dosing information)	Patient described as having a medically important condition. Respiratory distress was reported
3 months (4 kg)	Nonphysician health professional	Acetaminophen	Apnea, cyanosis, respiratory depression, and supratherapeutic drug levels were reported

## Abbreviations

AE: Adverse event
CNS: Central nervous system
FDA: Food and Drug Administration
GI: Gastrointestinal
NSAID: Nonsteroidal anti-inflammatory drug
RCT: Randomized controlled trial.
