# Supplementary material for: How Safe Are Common Analgesics for the Treatment of Acute Pain for Children? A Systematic Review
Source: Pain Res Manag. 2016 Dec 18;2016:5346819. doi: 10.1155/2016/5346819 (PMC5203901; doi:10.1155/2016/5346819)
Supplement: Supplementary file 1 — Appendix A: Search strategy for Medline. Appendix B: Detailed risk data by study and adverse event. Appendix C: This file contains study level details for each of the AEs described, including the dosage, the number of events, the sample size, and the risk. [file 5346819.f1.docx]

**Appendix A. Search Strategy***

* This appendix shows the search strategy for MEDLINE; other searches available from authors

Database: Medline via Ovid <1946 to April Week 4 2013>

Search: AEs in Acute Pain for Children 1.2 Outpatient setting | Medline – 9 May 2013 -- AM

Results: 1,059

| *MeSH and keywords for Pain:*  1. exp Pain/  2. exp Neuralgia/  3. Nociceptors/  4. pain*.mp.  5. oligoanalgesia.tw. |
| --- |
| 6. **or/1-5** [MeSH and pain*.mp] (537,806) |
| *MeSH and keywords for Outpatient Care:*  7. Acute Disease/  8. exp Ambulatory Care/  9. exp Ambulatory Care Facilities/  10. exp Ambulances/  11. Community Health Centers/  12. Emergencies/  13. Emergency Medical Technicians/  14. Emergency Medicine/  15. Emergency Service, Hospital/  16. Emergency Treatment/  17. exp Emergency Medical Services/  18. exp Outpatient Clinics, Hospital/  19. exp Primary Health Care/  20. ((accident* or casualt*) adj3 (service* or department* or room* or center* or centre* or unit*)).tw.  21. ((acute or emerg*) adj3 care).tw.  22. (ambulatory adj3 (service* or department* or room* or center* or centre* or unit*)).tw.  23. (community adj3 (service* or department* or room* or center* or centre* or unit*)).tw.  24. (ED or EDs or ER or ERs or EMS).tw.  25. ((emergenc* or emerg*) adj3 (department* or room* or ward* or unit* or care of hospital* or patient* or physician* or doctor* or treatment*)).tw.  26. (emergenc* adj3 (technician* or practitioner* or dispatch*)).tw.  27. (emergicenter* or emergicentre*).tw.  28. ((out-patient or outpatient) adj3 (service* or department* or room* or center* or centre* or unit*)).tw.  29. ((pain* or walkin or walk-in) adj3 (service* or department* or room* or center* or centre* or unit*)).tw.  30. ((prehospital or pre-hospital) adj3 (emergenc* or care*)).tw.  31. (primary adj3 care).tw.  32. (trauma adj3 (center* or centre*)).tw.  33. (urgent adj3 care).tw. |
| 34. **or/7-33** [MeSH and index terms for outpatient settings] (631,322) |
| *MeSH and keywords for Drugs of Interest:*  35. Acetaminophen/  36. exp Acetic Acid/  37. exp Analgesics, Opioid/  38. exp Anti-Inflammatory Agents, Non-Steroidal/  39. exp Fenamates/  40. exp Morphine/  41. Pain Management/  42. exp Propionates/  43. exp Salicylates/  44. exp Sulfonamides/  45. (Aspirin* or acetylsalicylic or ASA).mp.  46. Acetaminophen*.mp.  47. Acetic acid*.mp.  48. Acetylpropionylmorphine*.mp.  49. Alfentanil*.mp.  50. Allylprodine*.mp.  51. Alphamethylfentanyl*.mp.  52. Bezitramide*.mp.  53. Buprenorphine*.mp.  54. Butorphanol*.mp.  55. Carfentanyl*.mp.  56. Celecoxib*.mp.  57. Codeine*.mp.  58. ("COX-2 inhibitor*" or Coxib*).mp.  59. Desomorphine*.mp.  60. Dexibuprofen*.mp.  61. Dexketoprofen*.mp.  62. Dextromoramide*.mp.  63. Dextropropoxyphene*.mp.  64. Dezocine*.mp.  65. Diacetyldihydromorphine*.mp.  66. Dibenzoylmorphine*.mp.  67. Diclofenac*.mp.  68. Difenoxin*.mp.  69. Diflunisal.mp.  70. Dihydrocodeine*.mp.  71. Dihydroetorphine*.mp.  72. Diphenoxylate*.mp.  73. Dipipanone*.mp.  74. Dipropanoylmorphine*.mp.  75. Droxicam*.mp.  76. Enolic acid*.mp.  77. Ethylmorphine*.mp.  78. Etodolac*.mp.  79. Etoricoxib*.mp.  80. Etorphine*.mp.  81. Fenamate*.mp.  82. Fenamic acid*.mp.  83. Fenoprofen*.mp.  84. Fentanyl*.mp.  85. Firocoxib*.mp.  86. Flufenamic acid*.mp.  87. Flurbiprofen*.mp.  88. Heterocodeine*.mp.  89. Hydrocodone*.mp.  90. Hydromorphone*.mp.  91. Ibuprofen*.mp.  92. ("Levomethadyl Acetate*" or LAAM).mp.  93. Indomethacin*.mp.  94. Isoxicam*.mp.  95. Ketobemidone*.mp.  96. Ketoprofen*.mp.  97. Ketorolac*.mp.  98. Lefetamine*.mp.  99. Levomethorphan*.mp.  100. Levorphanol*.mp.  101. Licofelone*.mp.  102. Lipooxygenase*.mp.  103. Lornoxicam*.mp.  104. Loxoprofen*.mp.  105. Lumiracoxib*.mp.  106. Lysine clonixinate*.mp.  107. Meclofenamic acid*.mp.  108. Mefenamic acid*.mp.  109. Meloxicam*.mp.  110. Meperidine*.mp.  111. Meptazinol*.mp.  112. Methadone*.mp.  113. Methyldesorphine*.mp.  114. Morphine*.mp.  115. MPPP.mp.  116. Nabumetone*.mp.  117. Nalbuphine*.mp.  118. Naproxen*.mp.  119. Nicomorphine*.mp.  120. Nimesulide*.mp.  121. ("non-steroidal anti-inflammator*" or "nonsteroidal antiinflammator*").mp.  122. NSAID*.mp.  123. Ohmefentanyl*.mp.  124. opioid*.mp.  125. Opium*.mp.  126. Oripavine*.mp.  127. Oxaprozin*.mp.  128. Oxicam*.mp.  129. Oxycodone*.mp.  130. Oxymorphone*.mp.  131. papaveretum*.mp.  132. paracetamol*.mp.  133. Parecoxib*.mp.  134. Pentazocine*.mp.  135. PEPAP*.mp.  136. Pethidine*.mp.  137. Phenazocine*.mp.  138. Piritramide*.mp.  139. Piroxicam*.mp.  140. Prodine*.mp.  141. Propionic acid*.mp.  142. Propoxyphene*.mp.  143. Remifentanil*.mp.  144. Rofecoxib*.mp.  145. Salicylate*.mp.  146. Salsalate.mp.  147. Sufentanil*.mp.  148. Sulindac*.mp.  149. Sulphonanilide*.mp.  150. Tapentadol*.mp.  151. Tenoxicam*.mp.  152. Thebaine*.mp.  153. Tilidine*.mp.  154. Tolfenamic acid*.mp.  155. Tolmetin*.mp.  156. Tramadol*.mp.  157. Valdecoxib*.mp. |
| 158. **or/35-157** [MeSH and keywords for drugs of interest] (480,524) |
| *MeSH and keywords for Children:*  159. exp pediatrics/  160. exp Infant/  161. adolescent/  162. exp child/  163. (baby or babies or infant* or neonate* or toddler* or child* or adolescen* or teen* or youth or p?ediatric* or juvenile*).tw. |
| 164. **or/159-163** [MeSH and keywords for children] (3,014,687) |
| 165. **and/6,34,158,164** [pain + ED/ER + pain medications + children] (1,381) |
| 166. remove duplicates from 165 (1,352) |
|  |

**Appendix B. Results of Quality Assessment based on the McMaster Quality Assessment Scale of Harms***

| **Study** | **Harms Pre-defined** | **Serious AE defined** | **Severe AE defined** | **Mode of Collection ACTIVE** | **Mode of Collection PASSIVE** | **Who collected AE** | **Training/ background of assessors** | **Timing & Frequency of AE collection** | **Checklist Used for AE** | **Encompass all AE** | **No. of withdrawal and losses to follow-up specified** | **AE in each arm specified** | **No. and type of AE specified** | **Type of analysis** | **Score*** |
| --- | --- | --- | --- | --- | --- | --- | --- | --- | --- | --- | --- | --- | --- | --- | --- |
| Evers 2006 | No | No | No | Yes | Yes | Yes | No | Yes | No | Yes | Yes | Yes | Yes | No | 8 |
| Ruperto 2011 | No | No | No | Yes | Yes | Yes | Yes | Yes | No | Yes | Yes | Yes | Yes | No | 9 |
| Hamaleinen 1997 | No | No | No | Yes | No | Yes | No | Yes | No | Yes | Yes | Yes | Yes | Yes | 8 |
| Neri 2013 | No | No | No | No | Yes | Yes | Yes | No | No | Yes | Yes | Yes | Yes | Yes | 8 |
| Shepherd 2009 | Yes | No | No | Yes | Unsure | Yes | Yes | No | No | Yes | Yes | Yes | Yes | No | 8 |
| Drendel 2009 | Yes | No | No | Yes | Yes | Yes | Yes | Yes | Yes | Yes | Yes | Yes | Yes | No | 11 |
| Friday 2009 | Yes | No | No | Yes | No | No | No | No | No | Yes | Yes | Yes | Yes | No | 6 |
| Charney 2008 | Yes | No | No | Yes | No | Yes | No | No | No | Yes | Yes | Yes | Yes | Yes | 8 |
| Koller 2007 | No | No | No | No | Yes | Yes | Yes | No | No | Yes | Yes | Yes | Yes | Yes | 8 |
| Cukiemik 2007 | No | No | No | Yes | No | Yes | Yes | Yes | No | Yes | Yes | Yes | Yes | Yes | 9 |
| Clark 2007 | No | No | No | No | Yes | Yes | Yes | Unsure | No | Yes | Yes | Yes | No | Yes | 7 |
| Drendel 2006 | No | No | No | Yes | Yes | Yes | No | Yes | No | Yes | Yes | N/A | Yes | No | 7 |
| Wille 2005 | Yes | No | No | Yes | Unsure | Yes | Yes | Unsure | No | No | Yes | No | No | No | 5 |
| Lewis 2002 | Yes | No | No | Yes | No | Yes | Yes | Unsure | No | No | No | No | No | Yes | 5 |
| Tanabe 2002 | No | No | No | No | Unsure | Yes | Yes | No | No | Unsure | Yes | Yes | Yes | No | 5 |
| Bertin 1996 | Yes | No | No | Yes | Yes | No | Unsure | Yes | No | Yes | Yes | Yes | Yes | No | 8 |
| LeMay 2013 | Yes | No | No | Yes | No | Yes | Yes | Yes | Yes | Yes | Yes | Yes | Yes | No | 10 |
| Richer 2010 | No | No | No | Yes | No | Yes | Yes | No | No | Yes | No | N/A | Yes | No | 5 |
| Ismail 2007 | No | No | No | No | Yes | Yes | Yes | No | No | Yes | Yes | N/A | Yes | No | 6 |
| Soriani 2001 | No | No | No | No | Yes | Unsure | No | No | No | Yes | Yes | Yes | No | No | 4 |
| Pothmann 2000 | No | No | No | Unsure | Unsure | No | No | No | No | Unsure | No | Yes | Yes | No | 2 |
| Bertin 1991 | Yes | No | No | Yes | Yes | No | No | Yes | No | Yes | Yes | Yes | No | No | 7 |
| Poonai 2014 | No | No | No | Yes | No | Yes | No | Yes | No | Yes | Yes | Yes | Yes | Yes | 8 |

* The McHarms tool consists of 15 criteria, but we checked for only 14, as one (number of deaths) was not relevant to our study.

**Appendix C. Detailed Results**

**Appendix C - Table 1. Nausea reported in acute pain trials**

| **Author, Year** | | **Dosage** | **n/N** | **Risk (95% CI)** |
| --- | --- | --- | --- | --- |
| **Placebo** |  | | | |
| Hamalainen, 1997 | | NR | 3/81 | 0.04 [0.01, 0.10] |
| Total (placebo) | |  |  | **0.04 [0.01, 0.10]** |
| **Acetaminophen monotherapy** | |  |  |  |
| Hamalainen, 1997 | | 15mg/kg | 2/83 | 0.02 [0.01, 0.08] |
| Soriani, 2001 | | 15mg/kg | 3/33 | 0.09 [0.03, 0.24] |
| Cukiernik, 2007 | | 15mg/kg | 0/33 | 0.00 [0.00, 0.08] |
| Total (acetaminophen monotherapy) | | |  | **0.02 [0.00, 0.05]** |
| **Ibuprofen monotherapy** | | | | |
| Friday, 2009 | | 10mg/kg(400mg max) | 1/34 | 0.03 [0.01, 0.15] |
| Drendel, 2009 | | 10mg/kg | 9/169 | 0.05 [0.03, 0.10] |
| Hamalainen, 1997 | | 10mg/kg | 3/81 | 0.04 [0.01, 0.10] |
| Tanabe, 2002 | | 10mg/kg | 1/26 | 0.04 [0.01, 0.19] |
| Koller, 2007 | | 10mg/kg | 0/22 | 0.00 [0.00, 0.11] |
| LeMay, 2012 | | 10 mg/kg (max 600 mg) | 0/41 | 0.00 [0.00, 0.06] |
| Poonai, 2014 | | 10 mg/kg (max 600 mg) | 4/68 | 0.06 [0.02, 0.14] |
| Total (Ibuprofen monotherapy) | | |  | **0.03 [0.01, 0.05]** |
| **Naproxen** | |  |  |  |
| Cukiernik, 2007 | | 20mg/kg | 1/41 | 0.02 [0.00, 0.13] |
| Total (Naproxen) | |  |  | **0.02 [0.00, 0.13]** |
| **Nimesulide** | |  |  |  |
| Soriani, 2001 | | 2.5mg/kg | 2/33 | 0.06 [0.02, 0.20] |
| Total (Nimesulide) | |  |  | **0.06 [0.02, 0.20]** |
| **Morphine** | |  |  |  |
| Wille, 2005 | | 0.5mg/kg | 3/74 | 0.04 [0.01, 0.11] |
| Poonai, 2014 | | 0.5 mg/kg (max 10 mg) | 18/66 | 0.27 [0.18, 0.39] |
| Total (Morphine) | |  |  | **0.15 [0.00, 0.38]** |
| **Acetaminophen + Codeine** | |  |  |  |
| Drendel, 2009 | | 1 mg/kg per dose codeine component | 30/167 | 0.18 [0.13, 0.24] |
| Friday, 2009 | | 1mg/kg (60mg max) | 0/34 | 0.00 [0.00, 0.07] |
| Total (Acetaminophen + codeine) | | |  | **0.09 [0.00, 0.26]** |
| **Ibuprofen + Codeine** | |  |  |  |
| LeMay, 2012 | | Codeine = 1 mg/kg (max 60 mg); Ibuprofen = 10 mg/kg (max 600 mg) | 1/42 | 0.02 [0.00, 0.12] |
| Total (Ibuprofen + codeine) | |  |  | **0.02 [0.00, 0.12]** |
| **Oxycodone** | |  |  |  |
| Koller, 2007 | | 0.1mg/kg | 0/22 | 0.00 [0.00, 0.11] |
| Total (Oxycodone) | |  |  | **0.00 [0.00, 0.11]** |
| **Ibuprofen + Oxycodone** | |  |  |  |
| Koller, 2007 | | 10mg/kg+0.1mg/kg | 0/22 | 0.00 [0.00, 0.11] |
| Total (Ibuprofen + Oxycodone) | | |  | **0.00 [0.00, 0.11]** |

CI = confidence interval

**Appendix C - Table 2. Vomiting reported in acute pain trials**

| **Author, Year** | | **Dosage** | **n/N** | **Risk (95% CI)** |
| --- | --- | --- | --- | --- |
| **Placebo** |  | | | |
| Hamalainen, 1997 | | NR | 6/81 | 0.07 [0.03, 0.15] |
| Total (Placebo) | |  |  | **0.07 [0.03, 0.15]** |
| **Acetaminophen** | |  |  |  |
| Hamalainen, 1997 | | 15mg/kg | 2/83 | 0.02 [0.01, 0.08] |
| Drendel, 2009 | | 1 mg/kg per dose codeine component | 18/167 | 0.11 [0.07, 0.16] |
| Pothmann, 2000 | | 6-8yrs = 250mg, 9-12yrs = 500mg | 1/19 | 0.05 [0.01, 0.25] |
| Shepherd, 2009 | | 15mg/kg | 0/60 | 0.00 [0.00, 0.04] |
| Total (Acetaminophen) | |  |  | **0.04 [0.00, 0.09]** |
| **Ibuprofen** |  | | | |
| Drendel, 2009 | | 10mg/kg | 4/169 | 0.02 [0.01, 0.06] |
| Hamalainen, 1997 | | 10mg/kg | 4/81 | 0.05 [0.02, 0.12] |
| Shepherd, 2009 | | 10mg/kg | 2/40 | 0.05 [0.01, 0.17] |
| Friday, 2009 | | 10mg/kg(400mg max) | 0/34 | 0.00 [0.00, 0.07] |
| LeMay, 2012 | | 10 mg/kg (max 600 mg) | 0/41 | 0.00 [0.00, 0.06] |
| Poonai, 2014 | | 10 mg/kg (max 600 mg) | 2/68 | 0.03 [0.01, 0.10] |
| Total (Ibuprofen) | |  |  | **0.02 [0.00, 0.03]** |
| **Ketorolac** | |  |  |  |
| Neri, 2013 | | 0.5mg/kg(20mg max) | 0/64 | 0.00 [0.00, 0.04] |
| Total (Ketorolac) | |  |  | **0.00 [0.00, 0.04]** |
| **Tramadol** |  | | | |
| Neri, 2013 | | 2 mg/kg, to a max. of 100mg | 3/67 | 0.04 [0.02, 0.12] |
| Total (Tramadol) | |  |  | **0.04 [0.02, 0.12]** |
| **Morphine** | |  |  |  |
| Wille, 2005 | | 0.5mg/kg | 1/74 | 0.01 [0.00, 0.07] |
| Poonai, 2014 | | 0.5 mg/kg (max 10 mg) | 8/66 | 0.12 [0.06, 0.22] |
| Total (Morphine) | |  |  | **0.06 [0.00, 0.17]** |
| **Codeine** | |  |  |  |
| Charney, 2008 | | 2mg/kg | 1/56 | 0.02 [0.00, 0.09] |
| Total (Codeine) | |  |  | **0.02 [0.00, 0.09]** |
| **Acetaminophen + Codeine** | | | | |
| Friday, 2009 | | 1mg/kg (60mg max) | 1/34 | 0.03 [0.01, 0.15] |
| Total (Acetaminophen + Codeine) | | |  | **0.03 [0.01, 0.15]** |
| **Ibuprofen + Codeine** | |  |  |  |
| LeMay, 2012 | | Codeine = 1 mg/kg (max 60 mg); Ibuprofen = 10 mg/kg (max 600 mg) | 0/42 | 0.00 [0.00, 0.06] |
| Total (Ibuprofen + Codeine) | |  |  | **0.00 [0.00, 0.06]** |
| **Oxycodone** | |  |  |  |
| Charney, 2008 | | 0.2mg/kg | 1/51 | 0.02 [0.00, 0.10] |
| Total (Oxycodone) | |  |  | **0.02 [0.00, 0.10]** |

CI = confidence interval

**Appendix C - Table 3. Headache reported in acute pain trials**

| **Author, Year** | | **Dosage** | **n/N** | **Risk (95% CI)** |
| --- | --- | --- | --- | --- |
| **Acetaminophen** |  | | | |
| Cukiernik, 2007 | | 15mg/kg | 2/36 | 0.06 [0.02, 0.18] |
| Total (Acetaminophen) | |  |  | **0.06 [0.02, 0.18]** |
| **Naproxen** |  | | | |
| Cukiernik, 2007 | | 20mg/kg | 2/41 | 0.05 [0.01, 0.16] |
| Total (Naproxen) | |  |  | **0.05 [0.01, 0.16]** |
| **Codeine** | |  |  |  |
| Charney, 2008 | | 2mg/kg | 4/56 | 0.07 [0.03, 0.17] |
| Total (Codeine) | |  |  | **0.07 [0.03, 0.17]** |
| **Oxycodone** | |  |  |  |
| Charney, 2008 | | 0.2mg/kg | 6/51 | 0.12 [0.06, 0.23] |
| Total (Oxycodone) | |  |  | **0.12 [0.06, 0.23]** |

CI = confidence interval

**Appendix C - Table 4. Gastrointestinal symptoms* (excluding nausea and vomiting)**

| **Author, Year** | | **Dosage** | **n/N** | **Risk (95% CI)** |
| --- | --- | --- | --- | --- |
| **Placebo** |  | | | |
| Ruperto, 2011 | | 12mg/kg | 1/32 | 0.03 [0.01, 0.16] |
| Evers, 2006 | | NR | 1/29 | 0.03 [0.01, 0.17] |
| Hamalainen, 1997 | | NR | 0/81 | 0.00 [0.00, 0.03] |
| Total (Placebo) | |  |  | **0.00 [0.00, 0.02]** |
| **Acetaminophen monotherapy** | |  |  |  |
| Hamalainen, 1997 | | 15mg/kg | 0/83 | 0.00 [0.00, 0.03] |
| Cukiernik, 2007 | | 15mg/kg | 0/33 | 0.00 [0.00, 0.08] |
| Ismail, 2007 | | 500mg | 1/1 | 1.00 [0.27, 1.00] |
| Ruperto, 2011 | | 12mg/kg | 0/32 | 0.00 [0.00, 0.08] |
| Total (Acetaminophen monotherapy) | |  |  | **0.03 [0.00, 0.09]** |
| **Ibuprofen monotherapy** | |  |  |  |
| Drendel, 2009 | | 10mg/kg | 4/169 | 0.02 [0.01, 0.06] |
| Hamalainen, 1997 | | 10mg/kg | 1/81 | 0.01 [0.00, 0.07] |
| Evers, 2006 | | 200mg (for children <12) or 400mg (for adolescents) | 7/29 | 0.24 [0.12, 0.42] |
| Koller, 2007 | | 10mg/kg | 0/22 | 0.00 [0.00, 0.11] |
| Poonai, 2014 | | 10 mg/kg (max 600 mg) | 1/68 | 0.01 [0.00, 0.08] |
| Total (Ibuprofen monotherapy) | |  |  | **0.02 [0.00, 0.05]** |
| **Naproxen** | |  |  |  |
| Cukiernik, 2007 | | 20mg/kg | 1/41 | 0.02 [0.00, 0.13] |
| Total (Naproxen) | |  |  | **0.02 [0.00, 0.13]** |
| **Ketoprofen** | |  |  |  |
| Ruperto, 2011 | | 40mg | 0/33 | 0.00 [0.00, 0.03] |
| Total (Ketoprofen) | |  |  | **0.00 [0.00, 0.03]** |
| **Morphine** | |  |  |  |
| Poonai, 2014 | | 0.5 mg/kg (max 10 mg) | 4/66 | 0.06 [0.02, 0.15] |
| Total (Morphine) | |  |  | 0.06 [0.02, 0.15] |
| **Codeine** | |  |  |  |
| Charney, 2008 | | 2mg/kg | 17/56 | 0.30 [0.20, 0.43] |
| Total (Codeine) | |  |  | **0.30 [0.20, 0.43]** |
| **Acetaminophen + codeine** | |  |  |  |
| Drendel, 2009 | | 1mg/kg per dose codeine component | 3/167 | 0.02 [0.01, 0.05] |
| Total (Acetaminophen + codeine) | |  |  | **0.02 [0.01, 0.05]** |
| **Oxycodone** | |  |  |  |
| Koller, 2007 | | 0.1mg/kg | 0/22 | 0.00 [0.00, 0.11] |
| Charney, 2008 | | 0.2mg/kg | 13/51 | 0.25 [0.16, 0.39] |
| Total (Oxycodone) | |  |  | **0.12 [0.00, 0.37]** |
| **Ibuprofen + Oxycodone** | |  |  |  |
| Koller, 2007 | | 10mg/kg+0.1mg/kg | 1/22 | 0.05 [0.01, 0.22] |
| Total (Ibuprofen + Oxycodone) | |  |  | **0.05 [0.01, 0.22]** |

CI = confidence interval

*Gastrointestinal symptoms include diarrhea, gastric pain, pills become hard to swallow, felt sick, unwell with vomiting and

reduced urine output, emesis, constipation, sore stomach and abdominal pain, dry mouth and unspecified GI symptoms.

**Appendix C - Table 5. Drowsiness, sleepiness and tiredness**

| **Author, Year** | **Dosage** | **n/N** | **Risk (95% CI)** |
| --- | --- | --- | --- |
| **Acetaminophen** | | | |
| Shepherd, 2009 | 15mg/kg | 0/60 | 0.00 [0.00, 0.04] |
| Total (Acetaminophen) |  |  | **0.00 [0.00, 0.04]** |
| **Ibuprofen monotherapy** |  |  |  |
| Koller, 2007 | 10mg/kg | 3/22 | 0.14 [0.05, 0.33] |
| Shepherd, 2009 | 10mg/kg | 1/40 | 0.03 [0.00, 0.13] |
| LeMay, 2012 | 10 mg/kg (max 600 mg) | 0/41 | 0.00 [0.00, 0.06] |
| Poonai, 2014 | 10 mg/kg (max 600 mg) | 14/68 | 0.21 [0.13, 0.32] |
| Total (Ibuprofen monotherapy) | |  | **0.08 [0.00, 0.17]** |
| **Morphine** |  |  |  |
| Wille, 2005 | 0.5mg/kg | 18/74 | 0.24 [0.16, 0.35] |
| Poonai, 2014 | 0.5 mg/kg (max 10 mg) | 23/66 | 0.35 [0.24, 0.47] |
| Total (Morphine) |  |  | **0.29 [0.18, 0.40]** |
| **Codeine** |  |  |  |
| Charney, 2008 | 2mg/kg | 31/56 | 0.55 [0.42, 0.68] |
| Total (Codeine) | |  | **0.55 [0.42, 0.68]** |
| **Acetaminophen + Codeine** |  |  |  |
| Drendel, 2009 | 1mg/kg/ dose codeine component | 35/169 | 0.21 [0.15, 0.27] |
| Total (Acetaminophen + Codeine) | |  | **0.21 [0.15, 0.27]** |
| **Ibuprofen + Codeine** |  |  |  |
| Drendel, 2009 |  | 51/167 | 0.31 [0.24, 0.38] |
| LeMay, 2012 | Codeine = 1 mg/kg (max 60 mg); Ibuprofen = 10 mg/kg (max 600 mg) | 0/42 | 0.00 [0.00, 0.06] |
| Total (Ibuprofen + Codeine) | |  | **0.15 [0.00, 0.45]** |
| **Oxycodone** |  |  |  |
| Koller, 2007 | 0.1mg/kg | 1/22 | 0.05 [0.01, 0.22] |
| Charney, 2008 | 0.2mg/kg | 29/51 | 0.57 [0.43, 0.69] |
| Total (Oxycodone) |  |  | **0.31 [0.00, 0.82]** |
| **Ibuprofen + Oxycodone** |  |  |  |
| Koller, 2007 | 10mg/kg+0.1mg/kg | 3/22 | 0.14 [0.05, 0.33] |
| Total (Ibuprofen + Oxycodone) | |  | **0.14 [0.05, 0.33]** |

CI = confidence interval

**Appendix C - Table 6. Dizziness reported in acute pain trials**

| **Author, Year** | **Dosage** | **n/N** | **Risk (95% CI)** |
| --- | --- | --- | --- |
| **Acetaminophen** |  |  |  |
| Shepherd, 2009 | 15mg/kg | 0/60 | 0.00 [0.00, 0.04] |
| Total (Acetaminophen) |  |  | **0.00 [0.00, 0.04]** |
| **Ibuprofen monotherapy** |  |  |  |
| Drendel, 2009 | 10mg/kg | 4/169 | 0.02 [0.01, 0.06] |
| Shepherd, 2009 | 10mg/kg | 1/40 | 0.03 [0.00, 0.13] |
| Koller, 2007 | 10mg/kg | 0/22 | 0.00 [0.00, 0.11] |
| LeMay, 2012 | 10 mg/kg (max 600 mg) | 0/41 | 0.00 [0.00, 0.06] |
| Poonai, 2014 | 10 mg/kg (max 600 mg) | 6/68 | 0.09 [0.04, 0.18] |
| Total (Ibuprofen monotherapy) |  |  | **0.02 [0.00, 0.04]** |
| **Morphine** |  |  |  |
| Poonai, 2014 | 0.5 mg/kg (max 10 mg) | 8/66 | **0.12 [0.06, 0.22]** |
| Total (Morphine) |  |  | **0.12 [0.06, 0.22]** |
| **Codeine** |  |  |  |
| Charney, 2008 | 2mg/kg | 6/56 | 0.11 [0.05, 0.21] |
| Total (Codeine) |  |  | **0.11 [0.05, 0.21]** |
| **Acetaminophen + Codeine** |  |  |  |
| Drendel, 2009 | 1mg/kg per dose codeine component | 9/167 | 0.05 [0.03, 0.10] |
| Total (Acetaminophen + Codeine) |  |  | **0.05 [0.03, 0.10]** |
| **Ibuprofen + Codeine** |  |  |  |
| LeMay, 2012 | Codeine = 1 mg/kg (max 60mg); Ibuprofen = 10 mg/kg (max 600mg) | 0/42 | 0.00 [0.00, 0.06] |
| Total (Ibuprofen + Codeine) |  |  | **0.00 [0.00, 0.06]** |
| **Oxycodone** |  |  |  |
| Charney, 2008 | 0.2mg/kg | 10/51 | 0.20 [0.11, 0.32] |
| Koller, 2007 | 0.1mg/kg | 0/22 | 0.00 [0.00, 0.11] |
| Total (Oxycodone) |  |  | **0.09 [0.00, 0.28]** |
| **Ibuprofen + oxycodone** |  |  |  |
| Koller, 2007 | 10mg/kg+0.1mg/kg | 1/22 | 0.05 [0.02, 0.14] |
| Total (Ibuprofen + oxycodone) |  |  | **0.05 [0.02, 0.14]** |

CI = confidence interval

**Appendix C - Table 7. Dermatological symptoms (itchiness, rash, pruritus)**

| **Author, Year** | **Dosage** | **n/N** | **Risk (95% CI)** |
| --- | --- | --- | --- |
| **Placebo** |  |  |  |
| Ruperto, 2011 | 12mg/kg | 0/32 | 0.00 [0.00, 0.08] |
| Total (Placebo) |  |  | **0.00 [0.00, 0.08]** |
| **Acetaminophen** |  |  |  |
| Ruperto, 2011 | 12mg/kg | 0/32 | 0.00 [0.00, 0.08] |
| Total (Acetaminophen) |  |  | **0.00 [0.00, 0.08]** |
| **Ibuprofen monotherapy** | | | |
| Koller, 2007 | 10mg/kg | 1/22 | 0.05 [0.01, 0.22] |
| Friday, 2009 | 10mg/kg(400mg max) | 0/34 | 0.00 [0.00, 0.07] |
| Total (Ibuprofen monotherapy) |  |  | **0.01 [0.00, 0.04]** |
| **Ketoprofen** |  |  |  |
| Ruperto, 2011 | 40mg | 1/33 | 0.03 [0.01, 0.15] |
| Total (Ketoprofen) |  |  | **0.03 [0.01, 0.15]** |
| **Codeine** |  |  |  |
| Charney, 2008 | 2mg/kg | 11/56 | 0.20 [0.11, 0.32] |
| Total (Codeine) |  |  | **0.20 [0.11, 0.32]** |
| **Acetaminophen + Codeine** |  |  |  |
| Friday, 2009 | 1mg/kg (60mg max) | 1/34 | 0.03 [0.01, 0.15] |
| Total (T3) |  |  | **0.03 [0.01, 0.15]** |
| **Oxycodone** |  |  |  |
| Charney, 2008 | 0.2mg/kg | 7/51 | 0.14 [0.07, 0.26] |
| Koller, 2007 | 0.1mg/kg | 0/22 | 0.00 [0.00, 0.11] |
| Total (Oxycodone) |  |  | **0.06 [0.00, 0.20]** |
| **Ibuprofen + Oxycodone** |  |  |  |
| Koller, 2007 | 10mg/kg+0.1mg/kg | 2/22 | 0.09 [0.03, 0.28] |
| Total (Ibuprofen + Oxycodone) |  |  | **0.09 [0.03, 0.28]** |

CI = confidence interval

**Appendix C - Table 8. Central Nervous System symptoms (lightheaded, agitation, twitchiness and unspecified CNS symptoms)**

| **Author, Year** | **Dosage** | **n/N** | **Risk (95% CI)** |
| --- | --- | --- | --- |
| **Placebo** |  |  |  |
| Evers, 2006 | NR | 2/29 | 0.07 [0.02, 0.22] |
| Total (Placebo) |  |  | **0.07 [0.02, 0.22]** |
| **Ibuprofen monotherapy** |  |  |  |
| Evers, 2006 | 200mg (for children <12y) or 400mg (for adolescents) | 1/29 | 0.03 [0.01, 0.17] |
| Koller, 2007 | 10mg/kg | 0/22 | 0.00 [0.00, 0.11] |
| Total (Ibuprofen monotherapy) | |  | **0.01 [0.00, 0.06]** |
| **Oxycodone** |  |  |  |
| Koller, 2007 | 0.1mg/kg | 0/22 | 0.00 [0.00, 0.11] |
| Total (Oxycodone) |  |  | **0.00 [0.00, 0.11]** |
| **Ibuprofen + Oxycodone** |  |  |  |
| Koller, 2007 | 10mg/kg+0.1mg/kg | 1/22 | 0.05 [0.01, 0.22] |
| Total (Ibuprofen + Oxycodone) | |  | **0.05 [0.01, 0.22]** |

CI = confidence interval

**Appendix C - Table 9. Pulmonary symptoms**

| **Author, Year** | | **Dosage** | **n/N** | **Risk (95% CI)** |
| --- | --- | --- | --- | --- |
| **Placebo** |  | | | |
| Ruperto, 2011 | | 12mg/kg | 1/32 | 0.03 [0.01, 0.16] |
| Total (Placebo) | |  |  | **0.03 [0.01, 0.16]** |
| **Acetaminophen** | |  |  |  |
| Ruperto, 2011 | | 12mg/kg | 0/32 | 0.00 [0.00 , 0.08] |
| Total (Acetaminophen) | |  |  | **0.00 [0.00 , 0.08]** |
| **Ketoprofen** | |  |  |  |
| Ruperto, 2011 | | 40mg | 1/33 | 0.03 [0.01, 0.15] |
| Total (Ketoprofen) | |  |  | **0.03 [0.01, 0.15]** |
| **Morphine** | |  |  |  |
| Wille, 2005 | | 0.5mg/kg | 1/74 | 0.01 [0.00 , 0.07] |
| Total (Morphine) | |  |  | **0.01 [0.00 , 0.07]** |
| **Codeine** | |  |  |  |
| Charney, 2008 | | 2mg/kg | 4/56 | 0.07 [0.03 , 0.17] |
| Total (Codeine) | |  |  | **0.07 [0.03 , 0.17]** |
| **Oxycodone** | |  |  |  |
| Charney, 2008 | | 0.2mg/kg | 2/51 | 0.04 [0.01, 0.13] |
| Total (Oxycodone) | |  |  | **0.04 [0.01, 0.13]** |

CI = confidence interval
